# Media Exposure Related to the PTSS During COVID-19 Pandemic: The Mediating Role of Risk Perception

**DOI:** 10.3389/fpsyt.2021.654548

**Published:** 2021-04-22

**Authors:** Yiqing Wang, Ling Jiang, Shuang Ma, Qinian Chen, Chengbin Liu, Farooq Ahmed, Muhammad Shahid, Xiaohua Wang, Jing Guo

**Affiliations:** ^1^School of Social Development and Public Policy, Beijing Normal University, Beijing, China; ^2^School of Management, Beijing University of Chinese Medicine, Beijing, China; ^3^School of Sociology, Huazhong University of Science and Technology, Wuhan, China; ^4^Department of Anthropology, Quaid-I-Azam University Islamabad, Islamabad, Pakistan; ^5^World Health Organization, Balochistan, Pakistan; ^6^School of Public Health, Peking University, Beijing, China

**Keywords:** media exposure, PTSS, risk perception, COVID-19, public health

## Abstract

**Objectives:** The objectives of this study are to assess the relationship between media exposure and post-traumatic stress symptoms (PTSS) and to highlight the underlying mechanisms mediated by risk perception.

**Methods:** This survey was conducted online in China from February 1st to February 10th, 2020. A total of 2,858 Chinese citizens aged ≥18 years from 31 provinces and autonomous regions were recruited to participate in a cross-sectional study. Self-report questionnaires were used to assess media exposure, PTSS, and risk perception.

**Results:** The prevalence of respondents with heightened PTSS scores was 22.2%. After controlling for covariates, media exposure (more than five times a day) was significantly and positively associated with a high level of PTSS (*B* = 4.11, *p* < 0.001), and risk perception (worry and severity) significantly mediated the relationship between media exposure and PTSS (all 95% CIs did not include 0).

**Conclusions:** Based on these findings, the frequency of media exposure was associated with PTSS. Risk perception (worry and severity) mediated the relationship between media exposure and PTSS. The mental health, particularly PTSS, of the general population should be closely monitored and “infodemics” should be combatted while addressing the COVID-19 outbreak; cognitive interventions may be promising.

## Introduction

The COVID-19 pandemic caused increasing mental health problems among the public worldwide. The majority of studies have revealed the psychological outcomes of disasters, including epidemic outbreaks (1, 2). These psychological outcomes include post-traumatic stress disorder (PTSD), depressive symptoms, generalized anxiety disorder (GAD), panic disorder (PD), etc., among which PTSD is the most common outcome (3). A recent review showed that the frequency of PTSS ranged from 7 to 53.8% in different groups of people during the COVID-19 outbreak (4). Thus, this negative psychological outcome should receive more attention and the risk factors for PTSD must be identified.

Previous studies suggested that direct exposure to public health emergencies or disasters was related to PTSD (5, 6). Healthcare workers and survivors directly exposed to SARS (severe acute respiratory syndrome) or MERS (Middle East respiratory syndrome) reported a higher level of PTSD (7–9). However, home quarantine was applied during the COVID-19 outbreak in China. Many people were not directly exposed to the COVID-19 but had more media contact, which was delivered large pandemic-related information, such as daily updates about surveillance and active cases provided by official departments on websites and social media (10). Previous studies suggested exposure to trauma events through media could lead to PTSS (11, 12). However, a nationwide post-9/11 survey of primary care patients suggested that indirect exposure through media was not associated with PTSD (13). Furthermore, to our knowledge, no studies have examined the relationship between indirect exposure through media and PTSS during the COVID-19 outbreak. And less is known about the mechanisms that translate media exposure to PTSS through risk perception.

### Media Exposure to COVID-19 and PTSS

According to the risk factor model of disaster adjustment, disaster exposure is the primary factor affecting mental health and physical functioning after traumatic events (14). Disaster-related media exposure, one of the disaster exposures, can also lead to negative mental health outcomes (12, 15). A recent study suggested that the frequency of COVID-19-related social media exposure was significantly and positively correlated with depression and anxiety within the general population in China (16).

PTSS was always related to exposure to traumatic events. The COVID-19 pandemic, as a public health emergency and trauma event for people, was featured by its high contagiousness, the relatively high mortality rate, and uncertainty about the future. When faced with such a relatively high degree of change in society, people often dependent on media to search for information for guidance (17). However, excessive disclosure of epidemic information led to an “infodemic,” a term recently defined by the World Health Organization (WHO) as an “overabundance of information—some accurate and some not—that makes it hard for people to find trustworthy sources and reliable guidance when they need it” (18). Some previous studies reported an association between media exposure and PTSD (12, 19, 20). Recent studies about the COVID-19 found that exposure to the pandemic through media could lead to negative psychological outcomes (21–23). Therefore, it can be inferred that pandemic-related media exposure could predict PTSS.

### The Mediating Role of Risk Perception

Risk perception refers to people's judgments and evaluations of hazards to which they are or might be exposed (24), and mainly includes familiarity, control, and severity of the risk (25, 26). According to the cultivation theory proposed by George Gerbner, the formation of perceptions and beliefs about the real world is a result of media exposure (27). Media systems dependency theory also asserts that the media system may increase the effects on cognitive and behavioral change by providing unique and central information (17). These two theories suggest that media exposure is an important factor contributing to people's perceptions. Previous studies showed that exposure to disasters or pandemics through media could result in high-risk perception (28, 29). For example, in a study on Ebola virus disease (EVD) in the United States, frequent exposure to risk-elevating messages may increase people's perception of the risk of EVD (30). In recent studies about media use during the COVID-19 pandemic, having a high frequency of exposure to COVID-19 information on social media was associated with a higher risk perception for the pandemic (31, 32). In the initial stage of the pandemic, media disclosure was full of information about the number of infections and deaths without effective treatment. Thus, pandemic-related media exposure can be seen as an important source of high-risk perception. A report from the WHO indicates that reducing the duration and frequency of watching, reading, or listening to news related to COVID-19 is an important way to minimize fears (33). Thus, studies investigating the effect of the frequency of media exposure are important.

Moreover, risk perception plays a significant role in the development of mental health (34, 35). For example, ample studies have shown that the perceived severity of COVID-19 positively predicted mental health problems (36), According to the cognitive-behavioral model of stress, the health consequences of an environmental stressor depend on the appraisal of the threat (primary appraisal) and of the personal resources to deal with it (secondary appraisal) (37). From this perspective, the risk perception related to the COVID-19 pandemic (primary appraisal) together with a sense of uncontrollability may lead to psychological stress responses. Some empirical studies have suggested that higher risk perceptions of the COVID-19 pandemic were related to severe PTSD symptoms (38, 39). Hence, it is reasonable to infer that risk perception could affect PTSS. Taken together, the hypothesis is that risk perception mediates the relationship between media exposure and PTSS. However, few studies have examined this mechanism.

This study aimed to assess the relationship between the frequency of media exposure and PTSS and to further determine whether risk perception mediates the effects of media exposure on PTSS in Chinese adults during the COVID-19 outbreak. The first hypothesis concerns people with more frequent exposure to COVID-19 through the media report a higher risk of PTSS. The second hypothesis relates to risk perception mediates the relationship between media exposure and PTSS.

## Methods

### Participants and Procedure

The current study was conducted online from February 1 to February 10, 2020, and the questionnaires were distributed and retrieved through a web-based platform (https://www.wjx.cn/app/survey.aspx). We recruited participants aged ≥18 years who were able to complete the questionnaires independently. Convenience sampling and snowball sampling were used. First, the questionnaire link was shared via the WeChat group and WeChat Moments (WeChat, a popular online communication tool in China) by researchers. Second, each subject in WeChat including the researcher's relatives, friends, and students from different ages, occupations, and provinces was asked to participate in this study. Third, people who completed the questionnaire or saw the link were asked to send our questionnaire weblink to more people. The survey took ~20–30 min. A total of 2,858 participants from 31 provinces in China participated in the current study. All participants were informed of the aim of the survey and provided consent before they completed the questionnaire. Participation was unconditionally voluntary. The Ethics Committee of Beijing Normal University approved the study (NO. SSDPP-HSC2020003).

### Measures

**Media exposure** was assessed by one question. Respondents were asked how often they read or viewed information about the COVID-19 pandemic through any type of media. This item was scored on a 4-point scale: more than 5 times a day, 3–5 times a day, 2 times a day, and 1 time a day or less.

**Risk perception** was measured using an 11-item self-designed scale based on the risk perception scale for the SARS epidemic developed by Xiaofei Xie et al. (40). The original scale included 3 factors, uncontrollability (6 items), worry (3 items), and possibility (2 items). In the present study, the factors were modified to ensure that the scale could be more suitable for this study after exploratory and confirmatory factor analysis. The scale was divided into 3 factors after exploratory factor analysis (KMO = 0.789, Bartlett's test χ^2^ = 6485.05, *p* < 0.001). Ranges of factor loadings were 0.587–0.813 for uncontrollability, 0.604–0.802 for worry, and 0.537–0.729 for severity. Confirmatory factor analysis indicated a good fit (χ^2^/df = 10.797, *p* < 0.001, RMSEA = 0.059, GFI = 0.979, AGFI = 0.955, NFI = 0.948, RFI= 0.909, CFI = 0.953). The first factor was uncontrollability. Five items assessed people's perception of the controllability of the pandemic, such as “Is COVID-19 controllable for society?” Responses were recorded on a 7-point scale ranging from 1 (completely controllable) to 7 (completely out of control). Cronbach's alpha was 0.76. The second factor was worry about COVID-19. Three items assessed people's level of concern about the pandemic, such as “For me, is COVID-19 worrying?” This question has four response categories ranging from 1 (totally indifferent) to 7 (very worrying). Cronbach's alpha was 0.59. The third factor was the nature or the severity of the epidemic. This dimension has 3 items, such as “Is the effect of the COVID-19 short-term or long-term?,” with seven response categories (1 = “Short-term” to 7 = “Long-term”). Cronbach's alpha was 0.61. The corrected intercorrelations values were presented in Table 1. The average score of each subscale was used, with higher scores indicating higher levels of uncontrollability, worry, and severity of the COVID-19.

**Table 1:** Observed correlations and corrected correlations among items in three factors.

**Uncontrollability**						**Worry**				**Severity**			
**Item**	**1**	**2**	**3**	**4**	**5**	**Item**	**1**	**2**	**3**	**Item**	**1**	**2**	**3**
1	1	0.57	0.29	0.40	0.37	1	1	0.59	0.58	1	1	0.31	0.30
2	0.50	1	0.37	0.48	0.66	2	0.46	1	0.42	2	0.24	1	0.37
3	0.25	0.32	1	0.35	0.31	3	0.45	0.32	1	3	0.23	0.29	1
4	0.35	0.42	0.30	1	0.57								
5	0.32	0.58	0.27	0.50	1								

*Observed correlations are presented below the diagonal, and corrected correlations above the diagonal*.

**PTSS** was assessed by the self-report PTSD Checklist for DSM-5 (PCL-5), estimating the severity of DSM-5-related PTSS symptoms experienced over the past month (41). The Chinese version of the original PCL-5 has been validated and is widely used in trauma-related research and practice (42). This scale includes 20 items, with 5 response categories ranging from 0 (not at all) to 4 (extremely). The sum score ranges from 0 to 80 points, with higher scores indicating a higher level of PTSS. The PCL-5 can determine a provisional diagnosis in two ways, (a) the presence of at least one re-experiencing symptom (items 1–5), one avoidance symptom (items 6–7), two negative alterations in cognition or mood symptoms (items 8–14) and two arousal symptoms (items 15–20), all rated 2 or higher. and (b) the sum of the total score over the cut-point score of 31 points.

The covariates listed below were measured. Demographic variables included age (≥18 years old), gender (male or female), ethnicity (Han or other), marriage (having no spouse or having a spouse), education (junior high school and below, high school/technical school, junior college, undergraduate, or post-graduate and above), and income (low, middle, or high income). According to previous studies (9, 43), health-related variables including prior mental health problems (yes, no), prior exposure to potential trauma (yes, no), direct exposure (yes, no), the occurrence of two-week illnesses (yes, no) were measured.

### Statistical Analyses

We used the SPSS-23 statistical package to perform all statistical analyses. Descriptive analyses were used to describe the characteristics of the samples and the prevalence of respondents with heightened PTSS scores. One-way ANOVA was used to examine the relationship between media exposure, risk perception, and PTSS. Model 4 of the PROCESS macro (version 2.16.2) (44) for SPSS was used to examine the direct effect of media exposure on PTSS and the mediating effect of risk perception on the above relationship. The bootstrapping method (5,000 bootstrapping samples) with 95% confidence intervals (CIs) was conducted to detect the significance of the effects (45). According to Hayes and Preacher, dummy coding is firstly needed. For media exposure, 3 dummy variables (2 times a day, 3–5 times a day, more than five times a day) are constructed, and the reference category is one time a day or less. Then, we examined the direct effect, omnibus indirect effect, and relative indirect effect. If the omnibus indirect effect is statistically significant, there is at least one relative indirect effect that is different from zero, which supports the conclusion that risk perception mediates the effect of media exposure on PTSS. All models were adjusted for age, gender, ethnicity, educational level, marital status, income, prior mental health problems, direct exposure, the occurrence of two-week illnesses, and prior exposure to potential trauma (experience of a traumatic event in the last year).

## Results

### Descriptive Analyses

Among the participants, 1,326 (46.4%) were male, 1,532 (53.6%) were female, and 91.9% of the subjects were between 18 and 50 years old; participants above 50 years of age contribute to merely 8.1% of the samples. 95.8% of the total participants belong to the Han ethnicity. Approximately half of the respondents had completed undergraduate studies, and more than 60% were married. The large majority (88.6%) had a middle to high-income level. When it comes to the health condition of participants, 14.6% of the participants had prior mental health problems, and 7.0% had a 2-week illness. As for the traumatic exposure, the proportion for participants out of prior traumatic exposures and direct exposure was 82.8 and 75.2% respectively. However, 82.3% of the participants are under indirect exposure through media, and more than half (56.3%) of them read epidemic information more than 5 times a day during the COVID-19 outbreak. Moreover, the prevalence of respondents with heightened PTSS scores was 22.2%. More details are reported in Table 2.

**Table 2:** Descriptive data on sociodemographics, media exposure, and PTSS.

**Variables**	**Groups**	**Frequency (%)**
Gender	Male	1,326 (46.4%)
	Female	1,532 (53.6%)
Age	18–25	691 (24.2%)
	26–30	645 (22.6%)
	31–40	891 (31.2%)
	41–50	400 (14.0%)
	>50	231 (8.1%)
Ethnicity	Han	2,738 (95.8%)
	Other	120 (4.2%)
Married	No	1,137 (39.8%)
	Yes	1,721 (60.2%)
Education	Junior high school and below	268 (9.4%)
	High school/technical school	387 (13.5%)
	Junior college	488 (17.1%)
	Undergraduate	1,257 (44.0%)
	Post-graduate and above	458 (16.0%)
Income	Middle and High	2,531 (88.6%)
	Low	327 (11.4%)
Prior mental health problems	Yes	418 (14.6%)
	No	2,440 (85.4%)
Prior exposure	Yes	492 (17.2%)
	No	2,366 (82.8%)
Direct exposure	Yes	709 (24.8%)
	No	2,149 (75.2%)
Two-week disease	Yes	201 (7.0%)
	No	2,657 (93.0%)
Media exposure	More than 5 times a day	1,608 (56.3%)
	3–5 times a day	762 (26.7%)
	2 times a day	259 (9.1%)
	1 time a day or less	229 (8.0%)
PTSS	Yes	635 (22.2%)

### Relationship Between Media Exposure, Risk Perception, and PTSS

Table 3 presents the results of the one-way ANOVA of the variables. People with different media exposure frequencies had a significantly different sense of uncontrollability about the COVID-19 (*F* = 2.739, *p* < 0.05). However, *post-hoc* tests (Scheffe) indicated that there was no significant difference in sense of uncontrollability across media exposure frequencies.

**Table 3:** One-way ANOVA between media exposure, risk perception, and PTSS.

	***n***	**Uncontrollability [*M* (*SD*)]**	**Worry [*M* (*SD*)]**	**Severity [*M* (*SD*)]**	**PTSS [*M* (*SD*)]**
**Media exposure**					
More than 5 times a day^a^	1,608	3.52 (1.25)	5.85 (1.10)	5.53 (1.17)	19.02 (18.32)
3–5 times a day^b^	762	3.41 (1.16)	5.45 (1.14)	5.29 (1.12)	14.33 (15.89)
2 times a day^c^	259	3.45 (1.12)	5.23 (1.12)	5.10 (1.15)	16.81 (17.36)
1 time a day or less^d^	229	3.33 (1.19)	4.91 (1.47)	4.87 (1.41)	13.77 (16.88)
*F*		2.739^*^	67.001^***^	29.605^***^	15.589^***^
*Post-hoc* tests (Scheffe)			a > b, c, d; b > c, d; c > d	a > b, c, d; b > d	a > d

*** *p < 0.001*,

* *p < 0.05; a>b, c, d means the score of group a is significantly higher than the score of group b, group c, and group d, respectively. b > c, d means the score of group b is significantly higher than the score of group c, and group d; c > d means the score of group c is significantly higher than the score of group d; b>d means the score of group b is significantly higher than the score of group d; a > d means the score of the group a is significantly higher than the score of group d*.

People with different media exposure frequencies had significantly different degree of worry about the COVID-19 (*F* = 67.001, *p* < 0.001). *Post-hoc* tests (Scheffe) indicated that people who read epidemic information more than five times a day had significant higher scores of risk perception (uncontrollability) than those who read 3–5 times a day (*M*_*d*_ = 0.40, 95% CI: 0.26, 0.55), two times a day (*M*_*d*_ = 0.62, 95% CI: 0.41, 0.84) and one time a day or less (*M*_*d*_ = 0.94, 95% CI: 0.72, 1.17). People who read epidemic information 3–5 times a day (*M*_*d*_ = 0.54 95% CI: 0.30, 0.78) and two times a day (*M*_*d*_ = 0.32 95% CI: 0.03, 0.61) had significant higher scores of risk perception (uncontrollability) than those who read one time a day or less.

People with different media exposure frequencies considered significantly different severity of the COVID-19 (*F* = 29.605, *p* < 0.001). *Post-hoc* tests (Scheffe) indicated that people who read epidemic information more than five times a day had significant higher scores of risk perception (severity) than those who read 3–5 times a day (*M*_*d*_ = 0.24, 95% CI: 0.09, 0.38), two times a day (*M*_*d*_ = 0.43, 95% CI: 0.21, 0.65) and 1 time a day or less (*M*_*d*_ = 0.66, 95% CI: 0.43, 0.89). People who read epidemic information 3–5 times a day (*M*_*d*_ = 0.42 95% CI: 0.18, 0.67) had significantly higher scores of risk perception (uncontrollability) than those who read one time a day or less.

And, there was a significant difference in PTSS scores when people had different media exposure frequencies. *Post-hoc* tests (Scheffe) indicated that people who read epidemic information more than five times a day had significantly higher PTSS scores than those who read one time a day or less (*M*_*d*_ = 0.5.25, 95% CI: 1.79, 8.71).

### Mediating Effect of Risk Perception on the Association Between Media Exposure and PTSS

**Figure 1:**
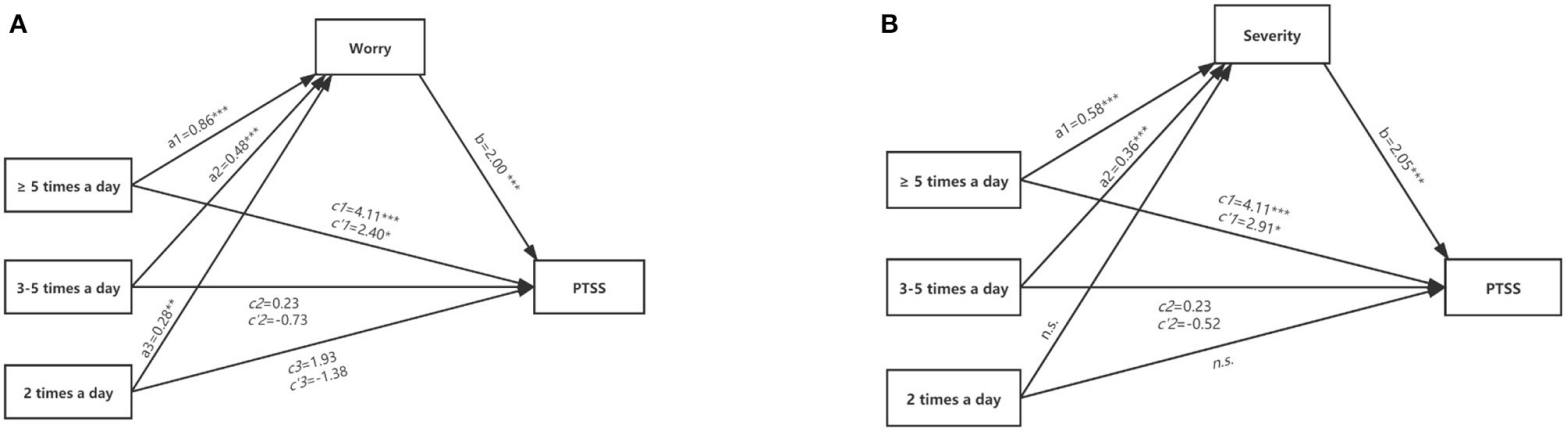
The mediation models of risk perception. **(A)** Depiction of the mediation model where risk perception (worry) mediates the relation between media exposure and PTSS; **(B)** Depiction of the mediation model where risk perception (severity) mediates the relation between media exposure and PTSS. a1-a3 and b, unstandardized coefficient; c'1-c'3, unstandardized coefficient after controlling mediators. n.s., the mediating effect of “2 times a day” on PTSS through “severity” was not significant. ***p < 0.001, **p < 0.01, *p < 0.05.

As shown in Table 4, media exposure (≥5 times a day) to COVID-19 was positively associated with PTSS (*B* = 4.11, *p* < 0.001). Table 4 also summarized the results of the mediation analysis. For the path through ‘uncontrollability’, the omnibus indirect effect and relative indirect effect were not statistically significant (95% CI include 0). For the path through “worry,” the omnibus indirect effect was significant (95% CI did not include 0), which indicated that there was at least one relative indirect effect that was significant. The relative indirect effect test suggested that all three relative indirect effects through “worry” were significant (95% CI did not include 0). Thus, compared to the reference group, the subgroup consuming COVID-19 media five times a day or more perceived as 0.86 units more worry about the COVID-19 (*a*_1_ = 0.86), the subgroup consuming COVID-19 media 3–5 times a day perceived as 0.48 units more worry (*a*_2_ = 0.48), and the subgroup consuming COVID-19 media 2 times a day perceived as 0.28 units more worry (*a*_3_ = 0.28). Furthermore, holding condition constant, those who perceived more worry about the COVID-19 also had higher PTSS scores (*b* = 2.00) (Figure 1A). The relative indirect effects of “worry” were *a*_1_*b* = 1.71; *a*_2_*b* = 0.96; *a*_3_*b* = 0.55.

**Table 4:** Mediating effects of risk perception on the relationship between media exposure and PTSS.

	**Direct effect (Relative total effects)**			**Omnibus mediation effect**		**Relative mediation effect**	
	**Coefficient**	***t***	***p***	**Indirect effect**	**95% CI**	**Indirect effect**	**95% CI**
**Direct path**							
D1 → PTSS	1.93	1.27	0.21				
D2 → PTSS	0.23	1.76	0.86				
D3 → PTSS	4.11	3.40^***^	** <0.001**				
**Path through uncontrollability**				0.001	[-0.005, 0.123]		
D1 → uncontrollability → PTSS						0.63	[−0.25, 1.51]
D2 → uncontrollability → PTSS						0.24	[−0.67, 1.15]
D3 → uncontrollability → PTSS						0.39	[−0.69, 1.47]
**Path through worry**				0.10	[0.07, 0.17]		
D1 → worry → PTSS						1.71	**[1.15, 2.40]**
D2 → worry → PTSS						0.96	**[0.51, 1.51]**
D3 → worry → PTSS						0.55	**[0.09, 1.11]**
**Path through severity**				0.05	[0.02, 0.08]		
D1 → severity → PTSS						1.19	**[0.75, 1.80]**
D2 → severity → PTSS						0.75	**[0.35, 1.30]**
D3 → severity → PTSS						0.38	[−0.094, 0.92]

*** *p < 0.001; 95% CI, 95% confidence intervals; D_1_, more than 5 times a day; D_2_, 3–5 times a day; D_3_, 2 times a day*.

*Covariates: age, gender, ethnicity, educational level, marital status, income, prior mental health problems, direct exposure, the occurrence of 2-week illnesses, and prior exposure to potential trauma*.

*Bold values indicates that the path was significant*.

Moreover, there was a significant omnibus indirect effect of media exposure on PTSS through “severity” (95% CI did not include 0). The relative indirect effect test indicated that two relative indirect effects were significant (95% CI did not include 0). Thus, compared to the reference group, the subgroup consuming COVID-19 media more than five times a day perceived as 0.58 units more severity of the COVID-19 (*a*_1_ = 0.58), the subgroup consuming COVID-19 media 3–5 times a day perceived as 0.36 units more severity (*a*_2_ = 0.36), Furthermore, holding condition constant, those who perceived more severity of the COVID-19 also had higher PTSS scores (*b* = 2.05) (Figure 1B). The relative indirect effects of “severity” were *a*_1_*b* = 1.19; *a*_2_*b* = 0.75.

## Discussion

This study aimed to examine the relationship between media exposure and PTSS and to study the mediating effects of risk perception on this relationship in Chinese adults during the COVID-19 outbreak. To our knowledge, this study is the earliest study to examine the mechanism underlying the relationship between media exposure and COVID-19-related PTSS in Chinese adults. This study found that the frequency of media exposure was associated with PTSS. This indicated compared to the reference group, the subgroup consuming COVID-19 media five times a day or more are more likely to have higher PTSS scores. Risk perception (worry and severity) mediated the relationship between media exposure and PTSS. Thus, the mental health, particularly PTSS, of the general population should be closely monitored and “infodemics” should be combatted while addressing the COVID-19 outbreak; cognitive interventions may be promising.

The prevalence of respondents with heightened PTSS scores was 22.2%. This prevalence was consistent with the studies about disasters. A systematic review indicated that the prevalence of PTSS in the general population ranged from 7 to 53.8% during the COVID-19 outbreak (4). Other studies assessed it ranged from 9.4 to 86.2% among the high exposed population within 3 months after the Wenchuan earthquake (46), and 14.6%-28.9% among the general population during the SARS outbreak in Canada (47, 48). In our samples, the prevalence of respondents with heightened PTSS scores was within these ranges. However, it should be cautious to compare the prevalence among different studies due to the various sample sizes, the difference in assessment methods, characteristics of the population, nature of the events, and the distance from the center of the emergencies. For example, a study assessing PTSS among the general population during the COVID-19 pandemic in China and using the same instruments as ours but different sample sizes suggested that the prevalence of PTSS was 4.6% (49). A systematic review reported the pooled prevalence of PTSS was 13% for healthcare workers, 27% for COVID-19 patients (50). Despite the differences, it is still important to pay attention to PTSS under the COVID-19 pandemic.

Consistent with our hypothesis, media exposure was significantly associated with increasing PTSS. People who read pandemic information more than 5 times a day have a higher level of PTSS than people who are exposed to the pandemic through the media 1 time a day or less. This result is consistent with the studies suggesting that media exposure to COVID-19 could result in negative mental health outcomes among general people (51, 52). According to the uncertainty reduction theory, people tend to seek information about the potential threat to reduce negative emotions (53), but exposure to distressing content through the media may increase people's stress for two reasons. First, in the initial stage of the great outbreak of COVID-19, there was an exponential increase in the number of infected and dead cases, causing large migration during Spring Festival. Meanwhile, the incubation period of the virus and its treatment were uncertain, and there was a possible asymptomatic transmission. Besides, stringent and large-scale quarantine measures were implemented in this stage. Therefore, most of the information from the media was the substantial increase in the number of infected cases and strict epidemic control measures, which led to panic among people and increased their risk of psychological trauma. Second, the information on the pandemic presented in the mass media was usually concrete and vivid, which was more likely to be remembered when people were exposed to this information frequently (20), which could reinforce rumination and intrusive thoughts, and activate fear circuitry (54, 55), thus increasing PTSS.

Risk perception (worry and severity) mediates the relationship between media exposure and PTSS. For the first path of the mediation process, the results suggested that media exposure was positively related to the perception of worry and severity about the COVID-19, which coincided with previous studies (56, 57). This finding also supported the cultivation theory (27) and media systems dependency theory (17), suggesting that media exposure was a factor for perceptions of people. Pandemic-related media coverage in the initial stage of the COVID-19 contained more information about the increase of infected cases or deaths. A previous study suggested that these information during the epidemic was more likely to lead to a high-risk perception in people (35). Besides, the COVID-19 is a highly contagious virus without effective treatment and adequate protective materials, which is more likely to lead to increased worry about the epidemic, thus resulting in mental health problems (40). Finally, according to the stress-coping model, people exposed to the COVID-19 through media perceive the pandemic as threatening and felt they had insufficient resources to cope with the threat, which resulting in health-related psychological distress, such as PTSS (37).

For the second path of the mediation process, risk perception (worry and severity) was positively related to PTSS, which in line with previous studies showing that increasing perceived worry and severity of the pandemic were positively associated with psychological problems (58–60). Worry is thought of as an increment of one's attention to the perceived threat. When the majority of attentional resources were allocated toward the potential threat, higher-order cognitions were unavailable to process trauma information in a flexible and integrative manner, thus may leading to PTSS (58). Additionally, social cognition theory suggests that individuals receive information from the environment and form stable cognition through the internal information processing process, thus affecting their development (61). From this perspective, with prolonged exposure to the pandemic through media, people will perceive more worry and severity over the pandemic after appraising the situation, which may result in PTSS.

However, the controllability and severity of the pandemic do not exert an indirect effect on the relationship between media exposure and PTSS. Specifically, participants' perceived uncontrollability was positively and significantly related to PTSS, which was consistent with previous indicating that perceived uncontrollability could lead to negative mental health outcomes, such as negative emotion, depression, anxiety, stress (62, 63). While exposure to the COVID-19 through media was not significantly associated with perceived uncontrollability, which indicated that media exposure could not significantly increase the perceived uncontrollability of the COVID-19 among Chinese people. A potential explanation for this finding is as following: Perceived worry and severity are more related to the negative effect of the COVID-19 on the physical and psychological health of individuals, quality of life of individuals, as well as social development. Perceived uncontrollability is more associated with the extent to avoiding being infected, and blocking the spread of the virus. After the outbreak, many measures to reduce the spread of the virus were disclosed by the media, including wearing a medical mask, frequent washing with soap and water, the lockdown of cities and schools, home quarantine, and travel bans (64–66). These effective measures indicated that the pandemic was not uncontrollable, thus media exposure could not increase perceived uncontrollability.

## Limitations

The current study has several limitations. First, the survey was cross-sectional, which did not allow for causal conclusions. People who experienced more trauma or distress may have reported greater exposure to distressing media content. This study did not measure whether within the same period respondents were experiencing other traumatic experiences (e.g., death of family members, etc.) which could have contributed to the high PTSS scores, more so than the media exposure. Future longitudinal studies are needed to attempt to address this issue. Second, we only adjusted for the effects of demographic characteristics to understand the association between media exposure, risk perception, and PTSS. However, many other potential factors (e.g., social support, psychiatric comorbidity, chronic disease, etc.) were not included in this study, and thus the results may be biased. Third, media exposure was assessed based on the frequency at which people read information about the pandemic. However, the media content (e.g., positive or negative news) and forms of media are also important factors contributing to mental health. Also, the variable media exposure captured was not differentiated qualitatively. Future studies are needed that focus on more detailed measurements of media exposure. Fourth, we recruited Chinese samples through convenient/snowball methods, elderly people and people who did not have a smartphone might be excluded, thus the representativeness of this sample to the general population may be biased. A more rigorous random sampling method or population-based survey could be implemented in the future to avoid these limitations. Fifth, the self-report method to investigate media exposure, risk perception, and PTSS may lead to subjective reporting bias. Thus, various measurements could be applied in the future to obtain more objective information.

### Implications

PTSS during the pandemic should receive more attention. Screening tests for PTSS should be provided after occurrences of the pandemic, and professional services should be delivered to the high-risk group. In the current study, risk perception affected PTSS related to the pandemic, indicating that efforts to change people's cognition could be promising, such as cognitive-behavioral therapy that would allow them to form rational or positive perceptions of COVID-19 and thus develop positive attitudes and behaviors to prevent mental health risks (67, 68).

Although a dispute exists about whether indirect exposure should be included in the DSM-5 (69), research has shown that people who are more exposed to COVID-19 through media are more likely to have a higher level of PTSS. Thus, social media management in emergency management is important, because “crisis spread” on social media that adds to people's “negative bias” makes the negative information impressive and widespread, which might increase the risk of PTSS. A higher frequency of media exposure resulted in high levels of PTSS through a mechanism mediated by risk perception. Therefore, efforts to disclose information about the pandemic objectively and truthfully and stop rumors promptly might be promising approaches that will allow people to analyze and perceive risk rationally and to reduce the risk of mental health problems.

## Conclusion

As shown in the current study, the prevalence of respondents with heightened PTSS scores after the COVID-19 outbreak was 22.2%. Exposure to the pandemic through media was associated with PTSS. People who read pandemic information more than five times a day have a higher level of PTSS than people who were exposed to the pandemic through the media one time a day or less. Moreover, exposure to the pandemic through the media may cause people to perceive more worry and severity about the pandemic, which leads to a high risk of PTSS. We hope that our findings will contribute to interventions related to media use and PTSS after the pandemic.

## Data Availability Statement

The raw data supporting the conclusions of this article will be made available by the authors, without undue reservation.

## Ethics Statement

The studies involving human participants were reviewed and approved by The Human Subjects Committee of School of Social Development and Public Policy, Beijing Normal University (Number: SSDPP-HSC2020003). The patients/participants provided their written informed consent to participate in this study.

## Author Contributions

YW and LJ were responsible for the analysis and interpretation of data, drafted the first version of the manuscript, and critically revised the manuscript. XW and JG designed this study and conceived this paper. SM, QC, CL, FA, and MS provided critical revisions of the manuscript for important intellectual content and approved the final manuscript. All authors approved the final manuscript as submitted and agree to be accountable for all aspects of the work.

## Conflict of Interest

The authors declare that the research was conducted in the absence of any commercial or financial relationships that could be construed as a potential conflict of interest.
